# Trophic niche partitioning between two prey and their incidental predators revealed various threats for an endangered species

**DOI:** 10.1002/ece3.8742

**Published:** 2022-03-18

**Authors:** Ève Rioux, Fanie Pelletier, Martin‐Hugues St‐Laurent

**Affiliations:** ^1^ Département de Biologie, Chimie et Géographie Centre for Northern Studies & Centre for Forest Research Université du Québec à Rimouski Rimouski Québec Canada; ^2^ Département de biologie Centre for Northern Studies Université de Sherbrooke Sherbrooke Québec Canada

**Keywords:** apparent competition, caribou, foraging, isotopic niche, resource partitioning, stable isotope

## Abstract

Documenting trophic niche partitioning and resource use within a community is critical to evaluate underlying mechanisms of coexistence, competition, or predation. Detailed knowledge about foraging is essential as it may influence the vital rates, which, in turn, can affect trophic relationships between species, and population dynamics. The aims of this study were to evaluate resource and trophic niche partitioning in summer/autumn between the endangered Atlantic‐Gaspésie caribou (*Rangifer tarandus caribou*) population, moose (*Alces americanus*) and their incidental predators, the black bear (*Ursus americanus*) and coyote (*Canis latrans*), and to quantify the extent to which these predators consumed caribou. Bayesian isotopic analysis showed a small overlap in trophic niche for the two sympatric ungulates suggesting a low potential for resource competition. Our results also revealed that caribou occupied a larger isotopic niche area than moose, suggesting a greater diversity of resources used by caribou. Not surprisingly, coyotes consumed mainly deer (*Odocoileus virginianus*), moose, snowshoe hare (*Lepus americanus*), and occasionally caribou, while bears consumed mainly vegetation and, to a lesser extent, moose and caribou. As coyotes and bears also feed on plant species, we documented trophic niche overlap between caribou and their predators, as searching for similar resources can force them to use the same habitats and thus increase the encounter rate and, ultimately, mortality risk for caribou. Although the decline in the Gaspésie caribou population is mostly driven by habitat‐mediated predation, we found evidence that the low level of resource competition with moose, added to the shared resources with incidental predators, mainly bears, may contribute to jeopardize the recovery of this endangered caribou population. Highlighting the trophic interaction between species is needed to establish efficient conservation and management strategies to insure the persistence of endangered populations. The comparison of trophic niches of species sharing the same habitat or resources is fundamental to evaluate the mechanisms of coexistence or competition and eventually predict the consequences of ecosystem changes in the community.

## Graphical abstract

We have reconstructed the trophic niches of two ungulate prey, moose and Atlantic‐Gaspésie caribou, and of two incidental predators, coyote and black bear, living in sympatry in a protected area surrounded by intensively disturbed habitats. Using stable isotopes, we showed a small overlap in dietary niche for the two sympatric ungulates, suggesting a low potential for resource competition. However, we highlighted a trophic niche overlap between caribou and their predators, which can increase the encounter rate and associated mortality risk for caribou, an endangered species according to the Canadian Species at Risk Act.

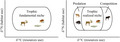

## 1 INTRODUCTION

Understanding ecological relationships among sympatric species is fundamental to evaluate underlying mechanisms of coexistence, competition, or predation, especially for species at risk that share common predators with an alternative prey (Holt, 1977). The persistence of prey species that are least productive is compromised by the exacerbated predation pressure exerted by predators that feed primarily on the most productive prey (DeCesare et al., 2010; Holt, 1984; Latham et al., 2011a, 2011b). However, coexistence of prey may be possible if less competitive prey avoid sectors and resources that are used by the most competitive prey (Holt, 1984) through the partitioning of their respective ecological niches (Latham, 1999).

**FIGURE 1:**
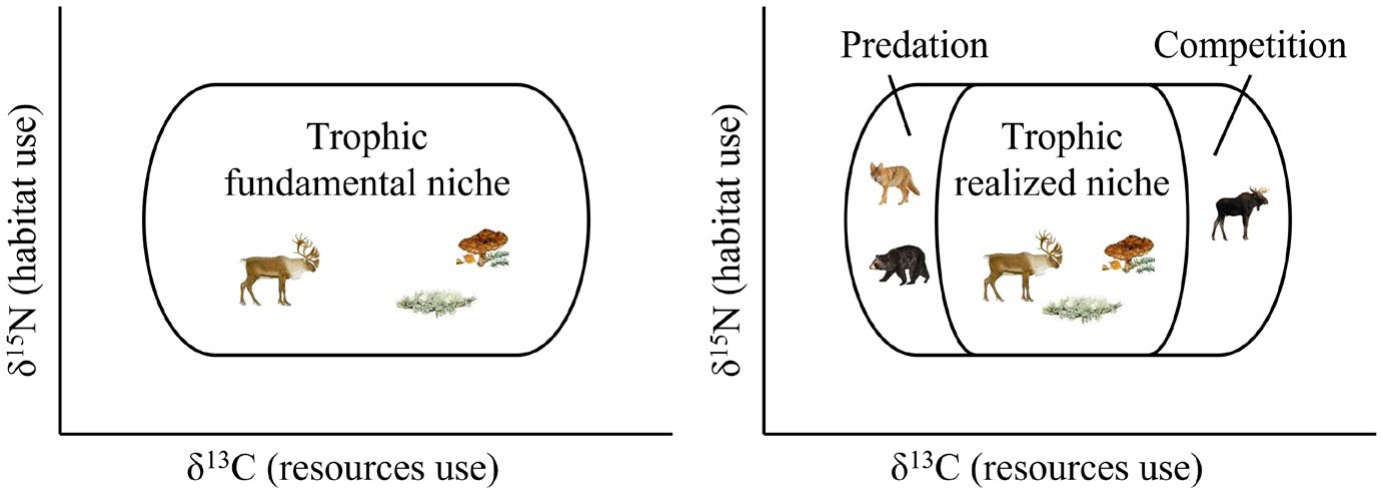
Schematic of the different trophic niche concepts presented in the bi‐dimensional isotopic space of δ^13^C (resources use) and δ^15^N values (habitat use). The trophic *fundamental niche* describes the range of optimal conditions wherein a species is able to persist in the absence of competition and predation, whereas the trophic *realized niche* considers all the constraints to which an animal is exposed to, including competition and predation (Hutchinson, 1957)

The ecological niche describes how a species interacts within an ecosystem and represents the interplay between biotic and abiotic variables that determine the conditions suitable for its survival, reproduction, and persistence (Hutchinson, 1957). The *fundamental ecological niche* describes the range of optimal conditions wherein a species is able to persist in the absence of competition and predation, whereas the *realized ecological niche* considers all the constraints to which an animal is exposed, including competition and predation (Figure 1) (Hutchinson, 1957). It was previously assumed that all individuals belonging to a given population were using the same niche, habitat, and resources (Hutchinson, 1957). However, there is increasing recognition that individuals are not identical and may have different feeding or habitat preferences leading to niche variation among individuals (Bolnick et al., 2003, 2007; Van Valen, 1965). The niche breadth is thus a trade‐off between the effect of intraspecific and interspecific competition for resources (Figure 1) (Roughgarden, 1972; Van Valen, 1965). If interspecific competition is low, intraspecific competition may trigger niche expansion by favoring the selection of novel resources (Lafferty et al., 2015), thereby reducing intraspecific competition, leading to an individual specialization (Bolnick et al., 2003) and allowing the coexistence of species (Jung et al., 2015; Latham, 1999). Coexistence or exploitative competition of sympatric species can occur due to resource partitioning in different habitats, according to different temporal activity patterns and under varying consumption levels of dietary sources (or prey size for predators; Latham, 1999; Schoener, 1974).

The trophic niche of an individual or a species belongs to the ecological niche, but it is built using a subset of variables related to trophic resources (Figure 1). The trophic niche may thus be described as the food resources selected and the foraging behaviors exhibited to acquire them (Araújo et al., 2011). It may be influenced by the location or time at which an animal forages (Robertson et al., 2015). Documenting the diet of wild species in different habitats is needed because it may help to better understand resource partitioning and trophic interactions. Indeed, foraging may affect individual fitness (Abramsky et al., 2002), vital rates (Parker et al., 1999), and population persistence (Macbeth & Kutz, 2019). However, a trade‐off between resource acquisition and predation avoidance can prevail. For example, in Yellowstone National Park, Hernández and Laundré (2005) showed that red deer (*Cervus elaphus*) moved from open meadows toward forest edges that provide lower‐quality forage but better protection from wolf (*Canis lupus*) predation. Characterizing the partitioning and level of overlap of trophic niches between prey and its conspecific competitors is critical to inform possible ecological relationships. This type of analysis can also be applied to prey that share resources with incidental, omnivorous predators which thus act simultaneously as predator and competitor. In such complex interactions, searching for similar resources in the same area can increase the encounter rate between prey and an opportunistic predator.

Diet composition is often inferred from the analysis of prey remains in scats and stomach contents of consumers (Lesmerises et al., 2015; Popp et al., 2018), but these techniques can be biased toward indigestible hard parts (McInnis et al., 1983; Nielsen et al., 2018). To overcome this limitation, DNA metabarcoding has emerged as a good option and provides high taxonomic resolution (Newmaster et al., 2013). However, these techniques only offer a snapshot of a consumer's diet (Lesmerises et al., 2015; Nielsen et al., 2018). In contrast, stable isotope analysis has become a key tool to study the foraging ecology of wild species as it provides long‐term information on diet assimilation (Kelly, 2000; Peterson & Fry, 1987). It is based on the principle that stable isotope ratios in the tissues of consumers reflect the ratios of their diet (DeNiro & Epstein, 1978, 1981). Changes in nitrogen stable isotope ratios (δ^15^N) occur from one trophic level to the next (+3–4‰), making them useful indicators of trophic position (Minagawa & Wada, 1984; Peterson & Fry, 1987; Post, 2002). In contrast, the carbon stable isotope ratio (δ^13^C) is particularly useful for delineating carbon sources and foraging locations (DeNiro & Epstein, 1981; Peterson & Fry, 1987). In addition, stable isotopes can provide insights into trophic niche ecology; as proposed by Newsome et al. (2007), the isotopic niche can be delineated as the bi‐dimensional isotopic space of δ^13^C and δ^15^N values in a bi‐plot (Figure 1). Isotopic niche analysis has been extended to assess how individuals or species partition food resources (Hobson et al., 2000), to better understand predator–prey relationships (Urton & Hobson, 2005) and interspecific competition (Jung et al., 2015), and to get insights into individual specialization (Newsome et al., 2009). For example, using stable isotope analysis, resource partitioning attributed to different diet selection (Merkle et al., 2017) and spatial segregation (Hobson et al., 2000) has been documented in three sympatric predators that coexist in North America; the gray wolf, grizzly bear (*Ursus arctos*), and black bear (*Ursus americanus*).

Most woodland caribou (*Rangifer tarandus caribou*) populations are declining in Canada and some small, isolated herds are particularly at risk (Festa‐Bianchet et al., 2011). This is the case of the Atlantic‐Gaspésie caribou population (hereafter referred as Gaspésie caribou population), the last herd of caribou found south of the St. Lawrence River. This herd is now considered endangered according to the Species at Risk Act (COSEPAC, 2014). Habitat‐mediated predation, exacerbated by habitat alteration, is identified as the main cause of population decline in several woodland caribou populations in Canada (Festa‐Bianchet et al., 2011) but also in the Gaspésie population (Frenette et al., 2020). In addition, intensive forest management occurring in the Gaspésie caribou habitat has led to a strong increase in moose (*Alces americanus*) density and was paralleled with an increase in density of black bears and coyotes (*Canis latrans*) (Frenette et al., 2020). These two incidental predators were shown to be the main predators of moose and caribou in the area (Crête & Desrosiers, 1995) but they also feed on a variety of plant species and smaller prey (Boisjoly et al., 2010; Mosnier et al., 2008). Although the decline in the Gaspésie caribou population appears mostly driven by habitat alteration due to forestry (Frenette et al., 2020), here we focus on the foraging ecology and the resource and trophic niche partitioning between the endangered Gaspésie caribou population, moose, and their two omnivorous predators. To do so, we used Bayesian stable isotopic analysis to reconstruct the diet composition of moose, coyotes, and bears, and to evaluate resource and trophic niche partitioning between caribou and these three species in the context of the apparent competition interaction (Holt, 1977). Such information is crucial to clarify the potential roles of interspecific resource competition between the endangered caribou population and moose and to determine to what extent these predators consumed (and even competed with) caribou. These omnivorous predators could theoretically enter into another type of competition (i.e., exploitation and/or interference competition) with caribou (and moose), at least for the plant species they share with both ungulates. By searching for similar food items, they could be forced to frequent similar habitat components (e.g., land‐ or forest‐cover types), which could increase encounter rate and ultimately the associated mortality risk.

## 2 METHODS

### 2.1 Study area

The study area is part of the southeastern boreal forest in the balsam fir (*Abies balsamea*) – white birch (*Betula papyrifera*) bioclimatic domain. It is located in the Gaspésie National Park and the surrounding Matane, Dunière, and Chic‐Chocs Wildlife Reserves, and Casault controlled harvesting zone (hereafter referred to as ZEC Casault) (Figure S1). The study area is characterized by three distinct vegetation zones distributed along the altitudinal gradient (Figure S1). The Gaspésie caribou population uses habitats found at high elevations (˃700 m; Mosnier et al., 2003). Population size has been declining for several decades, from 130 caribou in 1990 to ~40 individuals in 2019 (Morin & Lesmerises, 2020). A small fine‐scale genetic structure, with two subgroups (Logan‐Albert vs. McGerrigle), has been documented due to limited exchanges between summits (Figure S1; Pelletier et al., 2019). Intensive forestry activities conducted in the past decades have largely modified the landscape structure within and in the surroundings of the Gaspésie National Park, increasing the proportion of early‐seral forests to the detriment of mature coniferous forests (Boudreau, 2017). These changes have supported increases in moose density from 1.0 to 8.0 moose/10 km^2^ between 1992 and 2011 (Dorais, 2015). This increase was accompanied by an increase in bear and coyote densities (Frenette et al., 2020) that has exacerbated the predation pressure on caribou calves (Crête & Desrosiers, 1995) and adults (Lesmerises et al., 2019) via an apparent competition phenomenon (*sensu* Holt, 1977). This habitat‐mediated apparent competition is responsible for low calf recruitment rates (~8 calves per 100 females, Morin & Lesmerises, 2020) and low adult survival rates (77% for females and 56% for males in 2014 and 2015; Frenette et al., 2020).

### 2.2 Caribou, moose, and predator sample collection

We captured 44 caribou in the winter of 2013 and 2014 across the Gaspésie caribou range using a net gun fired from a helicopter. We collected caribou hair samples from the rump of the animal, and dried and stored them in paper bags at ambient temperature until processing. We used the same sampling and conservation protocol for hair samples collected from 90 moose, 127 coyote, and 57 black bear carcasses across the three Wildlife Reserves that overlap the Gaspésie National Park (Figure S1). Moose were harvested between September and October 2018 during the sport hunting season, whereas coyotes and bears were trapped during the annual predator control program between June 2016 and October 2018 across the Gaspésie caribou range. The capture and manipulation protocols were authorized by the Animal Welfare Committee [Université du Québec à Rimouski (hereafter UQAR) certificate #CPA‐52‐13‐112; Ministère des Forêts, de la Faune et des Parcs (hereafter MFFP) certificate #CPA FAUNE 13‐08].

### 2.3 Dietary source sample collection

We collected samples opportunistically from all potential food sources consumed by moose, coyotes, and bears to describe the composition of their diet. We collected hair samples from 22 individuals belonging to 6 different species that were accidentally trapped during the predator control program, including 4 white‐tailed deer (*Odocoileus virginianus*), 1 snowshoe hare (*Lepus americanus*), 2 large rodents (groundhog, *Marmota monax*, and North American porcupine, *Erethizon dorsatum*), 8 Canada lynx (*Lynx canadensis*), and 7 moose (*Alces americanus*). We also collected hairs from 22 individuals belonging to 6 species that were harvested by sport trappers or opportunistically collected in the ZEC Casault during summer, including 5 different species of rodents (1 red squirrel, *Tamiasciurus hudsonicus*, 2 common voles, *Microtus arvalis*, 5 deer mice, *Peromyscus maniculatus*, 6 Northern flying squirrels, *Glaucomys sabrinus*, and 1 pygmy shrew, *Sorex minutus*) and 1 species of large rodent (7 North American beavers, *Castor canadensis*). We also collected the feathers of three ruffed grouses (*Bonasa umbellus*) and hair from four snowshoe hares which were harvested by sport hunters in the ZEC Casault. We dried and stored hair and feathers in paper bags at ambient temperature until processing.

Finally, in the study area in 2017, we collected opportunistically six insects from three species belonging to the coleopteran (one beetle) and hymenoptera orders (three ants and two wasps). We also collected plant samples in July 2017 belonging to 46 different species in the montane boreal forest area of Mount Albert (*n* = 93) and Mount Logan (*n* = 72) in the Gaspésie National Park and of Petit Mount Ste‐Anne (*n* = 53) in the Chic‐Chocs Wildlife Reserves (see details in Appendix S1). Plant sampling was authorized by the Société des Établissements de Plein Air du Québec, which manages the Gaspésie National Park and the Chic‐Chocs and Matane Wildlife Reserves [certificate # PNG‐2017042703]. We randomly collected three replicates per species and froze them at −20°C until they were processed. We freeze‐dried insect and plant samples for 48 h, ground them into a fine powder using a CryoMill (Jardine et al., 2003), and stored them in a desiccator until the stable isotope analysis.

### 2.4 Hair and feather sample preparation

We washed hair samples using a solution of 2:1 chloroform–methanol in an ultrasonic bath to remove all possible surface contamination and external lipids, rinsed samples with distilled water, and oven‐dried them at 50°C for 24 h (Hobson et al., 2000). We freeze‐dried hair samples for 48h and ground them into a fine powder (Jardine et al., 2003). We used a CryoMill with a cooling system (liquid nitrogen at −196°C) for hair caribou samples only. For other animal samples, we cut the hairs and feathers into small pieces (about 1 mm) with stainless‐steel scissors and cleaned the scissors with ethanol 70% between samples.

### 2.5 Lipid extraction and stable isotope analyses

Stable isotope signatures measured in tissues may be biased due to the variability in the lipid content of samples because lipids are more depleted in ^13^C relative to protein and carbohydrate fractions (DeNiro & Epstein, 1977; McConnaughey & McRoy, 1979). We divided caribou hair samples into two parts to determine δ^13^C (lipid‐extracted) and δ^15^N (no lipid‐extracted) values separately to account for lipid effect on stable isotope signatures: one part of the subsamples received no further treatment prior to nitrogen isotope analysis, and the second part was lipid‐extracted prior to carbon isotope analysis (Kelly, 2000; Lesage et al., 2010; Post et al., 2007; Rioux et al., 2019). We conducted lipid extraction using the second part of powdered hair samples (to remove internal lipids) (Dunnett, 2005) and a solvent consisting of a mixture of chloroform and methanol (2:1 v/v) (Folch et al., 1957). We shook the mixture and stored it overnight at 4°C. We centrifuged the mixture at 11,200 g for 10 min and discarded the supernatant (Folch et al., 1957). We repeated the whole procedure twice. After three extractions, we dried samples by evaporation overnight, rinsed with distilled water, oven‐dried overnight at 50°C and powdered again. Due to methodological constraints, we used previously developed models of caribou normalization to correct the δ^13^C values of other animal hair for lipid content (equation 8 in Rioux et al., 2019).

We weighed 0.500–0.700 mg (± 0.001 mg) subsamples of powdered caribou hair and plant tissues, and 1.000–1.200 mg (± 0.001 mg) of other animal tissues and insects into a tin capsule. We analyzed samples to assess δ^13^C and δ^15^N using an elemental analyzer coupled to a delta plus continuous‐flow isotope ratio mass spectrometry. Analyses were conducted in the Marine Chemistry and Mass Spectrometry Laboratory (UQAR) for caribou hair and plant tissues, and in the Stable Isotope in Nature Laboratory (SINLAB, University of New Brunswick) for other animal tissues and insects. By convention, ^13^C and ^15^N isotope abundances are expressed in delta notation (‰), as δ*X* = [*(R*_sample_/*R*_standard_) −1] × 1000, where *X* is ^13^C or ^15^N, and *R*_sample_ is the corresponding ratio ^13^C/^12^C or ^15^N/^14^N; *R*_standard_ represents the ratios of the respective standards: Vienna Peedee Belemnite (PDB) and atmospheric nitrogen (AIR). We evaluated the accuracy of our isotopic analysis using three commercially certified materials (B2151, Acetanilide, and Nicotinamide) and the precision of measurement by randomly duplicating a subset of our samples. Replicates using certified B2151 materials (*n* = 31) indicated a systematic error of ±0.22 for δ^13^C and ±0.24‰ for δ^15^N, whereas replicates using certified Acetanilide and Nicotinamide materials (*n* = 36) indicated a systematic error of ±0.08‰ for δ^13^C and ±0.10‰ for δ^15^N. The average deviations observed between replicates of hair, fish muscle, insect, and plant samples (*n* = 97) indicated an analytical error of 0.16‰ for δ^13^C and 0.20‰ for δ^15^N.

### 2.6 Estimations of diet composition

Stable isotope ratios measured in hair reflect food consumption during the period of tissue growth (Schwertl et al., 2003); consequently, we assumed that the stable isotope signatures we calculated would represent the summer/autumn diet. For the species studied, molt occurs generally at the end of the cold season (April to June), and the new fur grows between late spring/early summer (June) to autumn (Darimont & Reimchen, 2002; Ling, 1970; Mowat et al., 2017). To estimate the relative contribution of the different food sources for the summer/autumn diets of moose, coyotes, and bears, we used the Bayesian stable isotope mixing model (hereafter referred to as SIMM) package in R (Parnell et al., 2013; Phillips et al., 2014). Diet composition for caribou was estimated in a companion study conducted in the same study area (Rioux et al., submitted). SIMMs have allowed the incorporation of variability in sources and trophic discrimination factors (hereafter referred to as TDFs), and the outputs represent true probability density functions (Moore & Semmens, 2008; Parnell et al., 2013; Phillips et al., 2014). Estimates are reported with their 95% credible intervals (hereafter referred to as 95% CI), which allow predicting within a 95% credibility level that source A represents from *x*% to *y*% of the assimilated diet (Parnell et al., 2013).

We included food sources known to be consumed by the species (moose, bears, and coyotes) as prior distributions into our SIMM and added prior distributions for each source (Moore & Semmens, 2008; Stock & Semmens, 2016) based on studies conducted in our study area (see details in Appendix S1). For moose, priors of diet composition came from Christopherson et al. (2019), who used DNA barcoding analysis on fecal pellets, while for bears and coyotes, we used data from fecal pellet analysis (M.‐H. St‐Laurent, *unpublished data*) (see details in Appendix S1). To facilitate source distinction in SIMM, we grouped plant samples into 10 functional groups: aquatic plants, deciduous trees, ericaceous shrubs, evergreen trees, ferns, forbs, fungi, horsetails, graminoids, and shrubs. Arboreal lichens, which represent most of the lichen biomass in our study area (Stone et al., 2008), were not considered as they are almost never consumed by moose, bears, and coyotes and because we were interested in describing trophic niche partitioning. We used the correlation matrix of food sources included in the SIMM package to verify the assumption of differences in isotopic signatures between sources. We combined the negatively correlated source proportions to gain precision in calculated proportions (Parnell et al., 2013; Phillips et al., 2014). We used the average TDFs (± SD) estimated by Rioux et al. (2020) for all the studied species during a controlled feeding trial carried out on 10 different individuals: 3 moose, 3 coyotes, and 4 black bears. Estimated TDFs reached 1.69 ± 0.93‰ for ∆^13^C and 4.86 ± 0.94‰ for ∆^15^N for moose, 3.41 ± 0.37‰ for ∆^13^C and 3.05 ± 0.13‰ for ∆^15^N for coyotes, and 5.92 ± 0.53‰ for ∆^13^C and 4.94 ± 0.47‰ for ∆^15^N for black bears (Rioux et al., 2020). Finally, we used a concentration‐dependent mixing model for bears and coyotes (Phillips & Koch, 2002) because there were considerable differences between carbon and nitrogen concentrations in plant and animal food sources (Table S1). Incorporating concentration dependence in the model ensures that the contribution of a source is proportional to the mass it contributes to the diet (Phillips & Koch, 2002). This model was not needed for moose as their diet consists only of plants. Model convergence was verified with Gelman–Rubin diagnostic tests, and the model was considered acceptable if values were <1.1 (Gelman et al., 2014).

### 2.7 Niche breadth and resource partitioning

We evaluated the niche breadth and food resource partitioning of our four focal species (caribou, moose, coyote, and bear) by estimating the Bayesian standard ellipse area (hereafter referred as SEA_B_) and the 95% CI in the bi‐dimensional isotopic space of δ^13^C and δ^15^N values using the SIBER library (Jackson, 2019; Jackson et al., 2011). The SEA_B_ contains 40% of the data and represents the core isotopic niche for each species in terms of the more frequent utilization of resources. The SEA_B_ is robust, less sensitive to extreme values or small sample sizes, and includes uncertainty around the community metrics (Jackson et al., 2011). We calculated the degree of niche trophic overlap among species with the overlap index of the SIBER model, where a value >1 indicates overlap between species. We also calculated the relative overlap proportion between species ellipses, where a value of 0 indicates no overlap and a value of 1 indicates complete overlap (Jackson et al., 2011). Finally, we also calculated the Layman metrics with the convex hull area to evaluate the degree of isotopic niche variability among individuals in the group (Layman et al., 2007). Convex hull is the smallest possible area that encompasses all points. It is highly sensitive to small sample sizes and extreme values contrary to SEA_B_ (Jackson et al., 2011), but our sample size was relatively large for each group of species. The total area of the convex hull (TA) represents the diversity of resources used by the species, while the mean distance to the centroid (CD) represents the dispersion and the diversity among consumers. We also calculated the mean nearest‐neighbor distance (NND), which is a measure of density and clustering within a group; it represents the niche habits of an individual compared to those of other individuals in the group. Finally, we calculated the standard deviation of NND (SDNND), which is a measure of evenness of isotopic space within a group. Based on Layman et al. (2007), the δ^13^C range represents a proxy of the diversity of resources supporting the consumers while the δ^15^N range represents a proxy of the vertical trophic structure of the population. We performed all statistical analysis using R software version 3.5 (R Development Core Team, 2017).

## 3 RESULTS

### 3.1 Diet composition estimates

**FIGURE 2:**
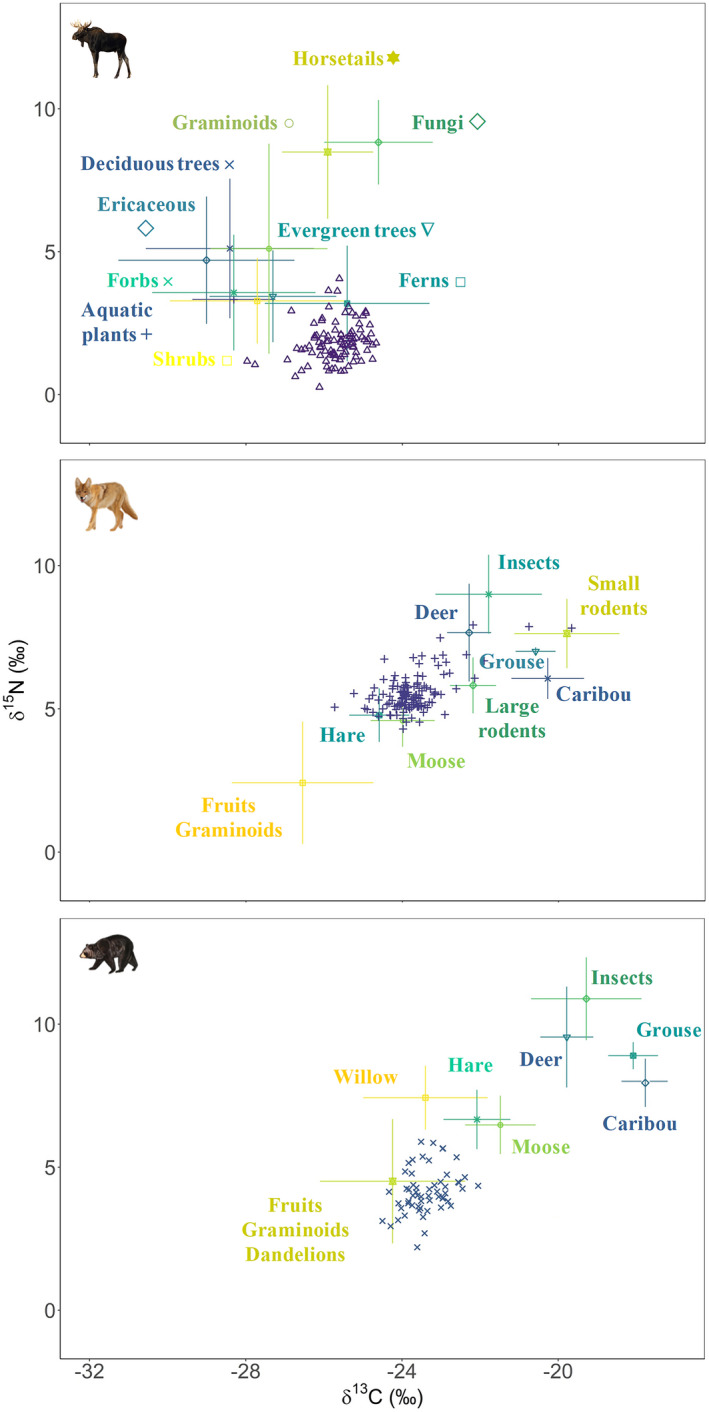
Carbon and nitrogen stable isotope signatures (mean ± SD) of dietary sources (solid points and error bars) and individual consumers (open circles ○) in the Gaspésie National Park and the surrounding area for moose, coyotes, and black bears

**FIGURE 3:**
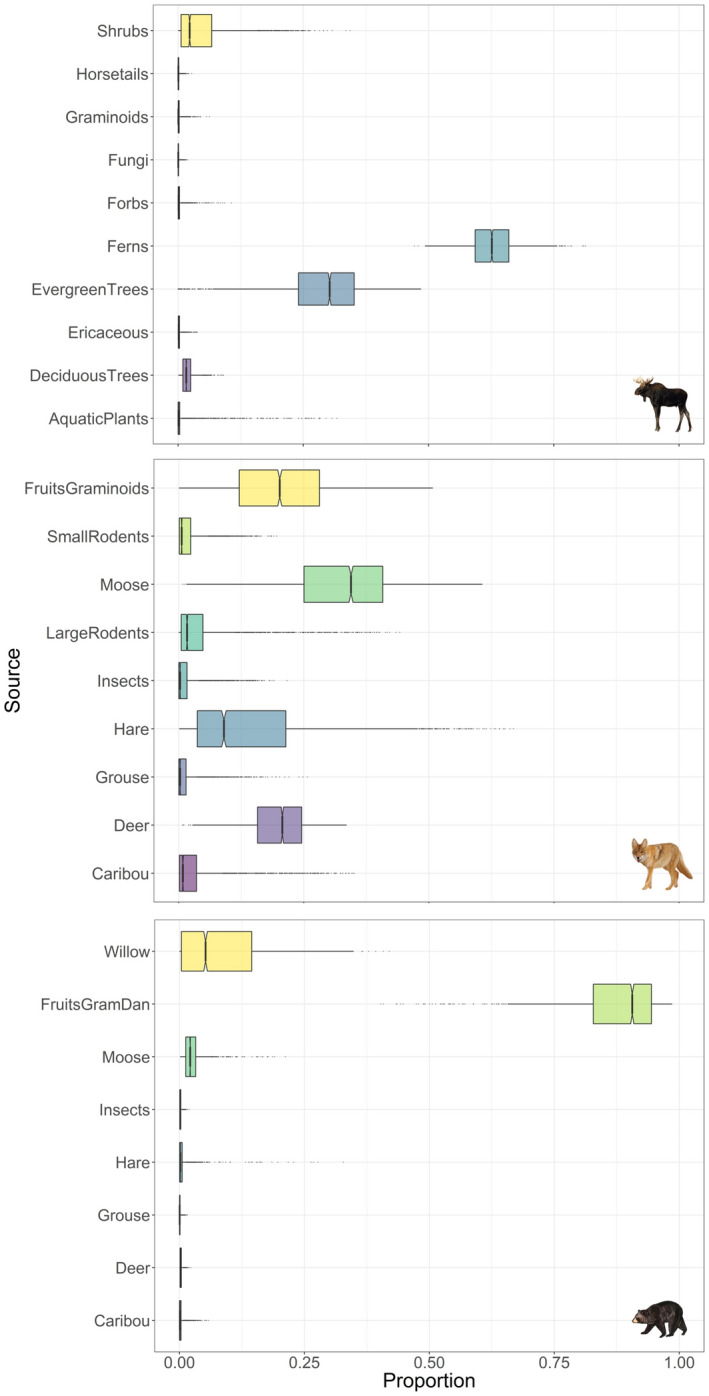
Proportional contributions of dietary sources (50, 75, and 95% CI) in the summer/autumn diet of moose, coyotes, and black bears using hair stable isotope ratios of carbon and nitrogen

Moose consumed mostly ferns (62.8% [54.1 to 72.8], mean [95% CI]), but also evergreen trees (28.8% [5.4 to 42.3]) and shrubs (4.6% [0.1 to 20.1]; Figures 2 and 3). Coyotes mostly had a carnivore diet and consumed deer, moose, and snowshoe hare (22.7% [13.5 to 29.2], 26.4% [8.3 to 44.3], and 27.5% [4.5 to 55.1], respectively; Figures 2 and 3). Coyotes occasionally consumed caribou (2.6% [0 to 11.1]) as well as fruits and graminoids (14.0% [5.4 to 24.7]; Figures 2 and 3). Finally, bears consumed mainly plants (Figures 2 and 3), such as dandelions (*Taraxacum* spp.), graminoids, fruits (89.4% [62.0 to 96.8]), and willow (5.4% [0.0 to 24.4]; Figures 2 and 3). In a lower proportion, they also consumed moose (3.1% [3.0 to 13.3]), hare (0.9% [0 to 4.7]), and caribou (0.3% [0 to 1.7], Figures 2 and 3).

### 3.2 Niche breadth and resource partitioning

**FIGURE 4:**
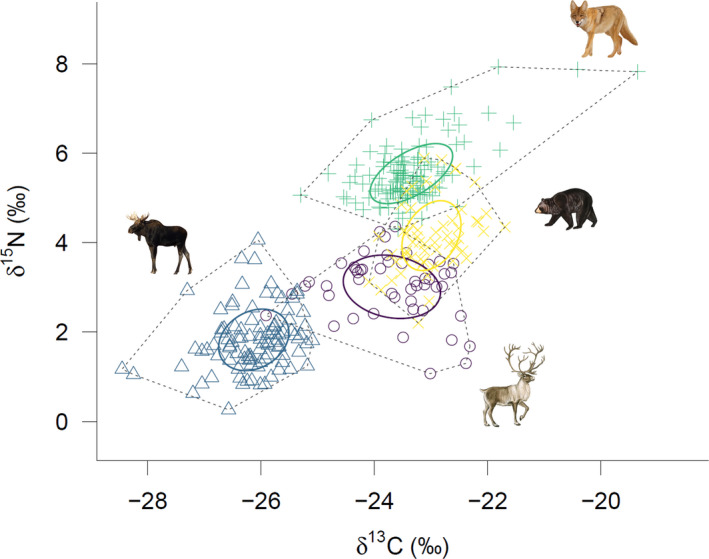
Isotopic niche areas in the bi‐dimensional isotopic space of δ^13^C and δ^15^N values of the four species (caribou, moose, coyote, and black bear) using SIBER. Circles show the standard ellipse areas (SEA_B_) and contain 40% of the data, while the dotted polygons show the convex hull areas (TA) and are drawn around the outermost points in the cloud of data

Isotopic niche area (SEA_B_) was larger for caribou (1.85‰^2^ [1.36–2.43], mode [95% CI]) than for moose (1.25‰^2^ [1.04–1.54]), coyotes (1.21‰^2^ [1.02–1.44]), and bears (1.18‰^2^ [0.89–1.53]) (Figure 4, Table 1). The probability of occupying a smaller isotopic niche area than caribou was 0.99 for all three species. The overlap index indicated that the caribou isotopic niche overlaps moose (1.29), coyote (1.25), and bear (4.19) niches. The isotopic niche overlap between coyotes and bears was 2.60. The relative overlap proportion between species ellipses was lower for the caribou vs. moose (0.07) and caribou vs. coyote (0.07) comparisons, but relatively higher between caribou vs. bear (0.23) and coyote vs. bear (0.18). The Layman metrics (Table 1) calculated with the convex hull area were larger for caribou (TA, CD, and NND reaching 0.95‰^2^, 0.91‰, and 1.36‰, respectively) than for moose, coyotes, and bears. SDNND was larger for moose (0.33‰) than for caribou, coyotes, and bears (Table 1). δ^15^N range was lower in coyotes compared to the three other species studied, while it was higher in caribou and bear groups (Figure 4, Table 1). δ^13^C range was higher in both cervid groups (Figure 4, Table 1).

**TABLE 1:** Layman metrics calculated from the convex hull areas and Bayesian standard ellipse area (SEA_B_) and their 95% credible intervals calculated from ellipse areas for caribou, moose, coyote, and black bear

Species	δ^15^N range (‰)	δ^13^C range (‰)	TA (‰^2^)	CD (‰)	NND (‰)	SDNND (‰)	SEA_B_ [95% CI] (‰^2^)
Caribou	1.30	1.66	0.95	0.91	1.36	0.08	1.85 [1.36–2.43]
Moose	0.66	1.48	0.38	0.55	0.54	0.33	1.25 [1.04–1.54]
Coyote	0.22	0.31	0.03	0.13	0.15	0.07	1.21 [1.02–1.44]
Black bear	0.84	0.32	0.15	0.33	0.38	0.02	1.18 [0.89–1.53]

## 4 DISCUSSION

Our study highlighted the partitioning of resources among caribou, moose, and their incidental predators and improved our understanding of their potential interactions. Using isotopic analysis, we found that only a few food sources were shared between caribou and moose and the overlap in their trophic niches appears low in summer/autumn. Our analyses also revealed a highly diversified diet for two omnivorous, opportunistic predators, including a low proportion of caribou and an overlap in their trophic niches. More interestingly, we reported a non‐neglectable trophic niche overlap between caribou and their predators (mainly black bears), suggesting that the quest for similar plant species can increase the encounter rate probability between these incidental predators and caribou, explaining partially the increased mortality risk and accelerated decline for this endangered population.

### 4.1 Resource partitioning between moose and caribou

Caribou consumed a great diversity of resources in summer/autumn, and we found a high interindividual difference in diet, potentially attributed to different niche habits. In a companion study (Rioux et al., submitted), we detailed caribou diet using the same tissue samples, and showed that caribou consumed mainly lichens, deciduous trees, and shrubs, but also mosses, evergreen trees, ferns, and horsetails. These results are supported by findings obtained by Christopherson et al. (2019) in the same study area where deciduous trees, shrubs, and horsetails were the main food source of Gaspésie caribou identified using the DNA barcoding analysis of their scats (note that lichens and fungi were not considered in their DNA barcoding analysis). For moose, our diet analysis indicated that the diversity of resources consumed was less diversified than for caribou and that diet is more homogenous between individuals, as seen with the narrower CI around source proportion estimates. In contrast, Christopherson et al. (2019) observed a higher diet Simpson diversity index, species richness, and food niche for moose compared to caribou. In our study, moose diet consisted mainly of ferns and, in lower proportion, of evergreen trees and shrubs, while DNA barcoding analysis of moose scats conducted by Christopherson et al. (2019) confirmed a high consumption of deciduous trees, evergreen trees, and shrubs, but they did not report consumption of ferns. However, consumption of ferns by moose is reported in other moose populations in Maine (Lautenschlager et al., 1997) and Alaska (Welch et al., 2015). As DNA barcoding analysis recorded a relatively shorter time window (~ day) compared to isotopic analysis in hair (~ months), this temporal difference in diet integration could explain the slight differences in diet composition observed between both studies.

Similar to Christopherson et al. (2019), we found a low potential for resource competition between caribou and moose in our study area, including the Gaspésie National Park and the surrounding Wildlife Reserves, as indicated by the small niche overlap between these two cervids. However, we cannot confirm that competition never occurred between these two sympatric cervids. In addition, consumers that feed on two resources with widely different isotopic compositions will always be found to have broader isotopic niches than animals that feed on food sources with less divergent δ‐values (Matthews & Mazumder, 2004; Newsome et al., 2007). Newsome et al. (2007) have suggested that the trophic niche breadth does not necessarily correspond to the diversity of the resources used because it also depends on the isotopic variability of these resources.

Some studies highlighted that resources partitioning between ungulates may be a result of past competition (caribou and moose: Christopherson et al., 2019; mule deer *Odocoileus hemionus*, moose, and elk: Hodder et al., 2013). Caribou and moose have lived in sympatry for a long time and coevolved to decrease exploitative competition, which may explain the different diet and the segregation of their trophic niches (Latham, 1999). Also, some studies documented spatial segregation of caribou from moose (Cumming et al., 1996; Seip, 1992), assuming that caribou select habitats less favorable to moose to reduce the encounter probability with predators that mostly focus on moose (Bergerud, 1985; James et al., 2004). This is assumed to be true in our study area, as these two cervids are thought to frequent different elevations in summer, with caribou selecting subalpine and alpine areas (>700 m) (Mosnier et al., 2003), while moose select mixed and early seral habitats found at lower elevations. Spatial segregation is known to allow coexistence, decrease exploitative competition, and reduce dietary overlap between species (Svanbäck & Bolnick, 2007). However, moose have more frequently been seen in the alpine tundra in summer in the Gaspésie National Park over the last 15 years, and an increase in the proportion of wintering grounds at high elevations (>600 m) was observed in winter (Roussel‐Garneau & Larocque, 2020) even though they prefer lower elevations with an abundance of early successional vegetative species. Spatial refuges of caribou may be compromised by high moose density and by the presence of moose in the alpine refuge habitat, potentially reducing access to highly nutritive resources and affecting physical and physiological conditions. Nutrition is known to contribute, at least partially, to the decline in this population as a secondary cause that predisposes females to poor reproductive performance and low calf survival rates (Rioux et al., submitted). The high moose density in the area is probably harmful to caribou because these two species share common predators.

### 4.2 Coexistence of generalist predators

We found moderate niche overlap between coyotes and bears. This niche segregation may drive the partitioning of the diet, allowing a better resource and habitat partitioning between these predators. This appears to facilitate coexistence by reducing potential competition between them. Indeed, the trophic niche width of both predators may indicate a great diversity of resources consumed and the wide 95% CI for certain food sources in the diet of coyotes, highlighting the generalist behavior of food selection or the influence of the local diversity of resources (Araújo et al., 2011; Bolnick et al., 2002).

In the boreal forest, coyotes depend mainly on human‐disturbed forests such as recent (5‐ to 20‐year‐old) clear‐cuts (Boisjoly et al., 2010). This disturbed habitat provides abundant fruit‐bearing shrubs (Brodeur et al., 2008), dense understory for snowshoe hares (St‐Laurent et al., 2008), and sufficient cover for moose (Dussault et al., 2005), which act as important food resources for coyotes (Boisjoly et al., 2010). As expected, we found that they had a carnivore diet and consumed mostly deer, moose, and hares. Moose and snowshoe hares are found in relatively high densities in the Gaspésie caribou range, which was reflected in the coyote's diet (around 26% for each food source). This result is also supported by coyote scat analysis conducted in Gaspésie (Boisjoly et al., 2010) and in the adjacent eastern New Brunswick (Dumond et al., 2001). We also found that coyotes consumed fruits and graminoids. Other studies have reported wild berry consumption by coyotes in Gaspésie (Boisjoly et al., 2010; Samson & Crête, 1997) and on the south shore of the St‐Lawrence River in southeastern Quebec (Tremblay et al., 1998). Coyotes consumed caribou occasionally, as previously reported in Gaspésie (Boisjoly et al., 2010; Crête & Desrosiers, 1995).

A companion study conducted in the Gaspésie caribou range showed that bears selected barren areas and mature coniferous forests in spring, and barren areas and 5‐ to 20‐year‐old clear‐cuts in summer and autumn, where abundant vegetation is found (Mosnier et al., 2008). As expected, we found that bears mostly consumed vegetation such as fruits, graminoids, dandelions, and willow. The diet of bears is closely linked to plant availability (Mosnier et al., 2008), and frequent interpatch movements between vegetation‐rich areas could result in a high encounter rate with moose and caribou neonates even without actively searching for them (Bastille‐Rousseau et al., 2011). We noted a low consumption of caribou and moose by bears (<3%) despite the very high moose density in the study area. Based on scat analysis, bears were shown to consume mostly vegetation (~95%) across the Gaspésie caribou range (Mosnier et al., 2008). A large consumption of fruits and graminoids allows bears to fulfill their daily energy requirements and allocate the remaining energy in fat reserves in anticipation of winter torpor.

### 4.3 Resource partitioning among caribou, coyotes, and bears

A novel aspect of our study refers to the empirical evidence of trophic niche overlap between caribou, an endangered ungulate prey that is a strict herbivore, and its incidental predators (coyotes and bears), which are opportunistic omnivores, suggesting that cross‐trophic competition might be at play between these three species. While previous studies have shown that caribou, coyotes, and bears are spatially distributed in different elevation zones (Mosnier et al., 2008), our results suggest that their respective diet and foraging strategies might increase encounter rates between them.

Bears and coyotes are important predators of moose, white‐tailed deer, and caribou calves (and to a lesser extent adults) in Québec (Bastille‐Rousseau et al., 2011; Leclerc et al., 2014) but also specifically in the Gaspésie National Park (Boisjoly et al., 2010; Crête & Desrosiers, 1995). To isolate themselves from predators, caribou are known to select higher elevations and mountain summits in Gaspésie (Mosnier et al., 2003, 2008), a spacing away strategy that allows caribou to reduce the risk of encounter, detection, and predation (Bergerud, 1985; James et al., 2004). However, intensive forest management has led to a marked decrease in the availability of mature fir stands rich in arboreal lichens (Stone et al., 2008), which were converted into early seral stages suitable to moose (Nadeau Fortin et al., 2016). Human‐driven habitat changes have been shown to support an increase in predator density (Boudreau, 2017) in response to an increase in small mammals (Etcheverry et al., 2005), fruit‐bearing shrubs (Boisjoly et al., 2010; Lesmerises et al., 2015), and moose densities (Frenette et al., 2020). Moreover, an important forest road network established to support forest management, the presence of hiking trails, and the movement capacities of predators facilitate coyote and bear dispersal into caribou habitat, especially in the alpine tundra where caribou are found during calving (Gaudry, 2013; Mosnier et al., 2005, 2008).

In such an altered landscape, avoiding predators might be more difficult now than it was before (1998–2004; Mosnier et al., 2008). The relative abundance of both coyotes and bears was shown to have a strong influence on caribou calf recruitment in Gaspésie, and their effect appears influenced not only by the relative abundance of moose but also by habitat modifications (Frenette et al., 2020). We went a step further by showing that the respective diet of caribou and its incidental predators, which also rely on plants, may force caribou to use the same habitats where common resources shared with predators (mostly with bears) can be found, thus explaining at least partially opportunistic predation on caribou calves.

### 4.4 Conclusions, limitations, and future research

Considering the low proportion of caribou found in the diet of predators in our study area, as well as the limitation of stable isotope analysis to detect scarce food sources (Nielsen et al., 2018; Phillips et al., 2014), more studies are needed to assess the diet composition of predators during other periods of the year, including the caribou neonatal stage when predation is most important (Crête & Desrosiers, 1995; Pinard et al., 2012). We also suggest that future research combines diverse dietary approaches in their analyses. Nevertheless, we found a low proportion of caribou in the diet of predators in our study area, which corresponds with their status of opportunistic predators. However, our study presented evidence suggesting that the omnivorous diet of bears and coyotes – and their trophic niche overlap with caribou – may play a key role in their predator–prey relationship with caribou. While coyotes and bears exert an incidental predation on caribou at the individual level, we consider that the high densities of these two predator species in our study area could explain the low recruitment noted for the endangered caribou in Gaspésie. In addition, we suggest that even a low level of food and habitat overlap with moose can contribute to the decline in this population or limit its potential to recover, given the precarity of the Gaspésie caribou population in the context of apparent competition interaction (Holt, 1977). Restoration (Lacerte et al., 2021) and protection of the last suitable habitat alongside other strategies like maternal penning, moose hunting, and predator control (Johnson et al., 2019) are needed to establish efficient conservation and management strategies to insure the persistence of this caribou population.

## CONFLICT OF INTEREST

The authors declare that there is no conflict of interest.

## AUTHOR CONTRIBUTIONS

**Ève Rioux:** Conceptualization (lead); Formal analysis (lead); Investigation (lead); Methodology (lead); Writing – original draft (lead). **Fanie Pelletier:** Conceptualization (equal); Formal analysis (supporting); Funding acquisition (supporting); Supervision (supporting); Writing – original draft (supporting); Writing – review & editing (equal). **Martin‐Hugues St‐Laurent:** Conceptualization (equal); Data curation (lead); Formal analysis (supporting); Funding acquisition (lead); Project administration (lead); Resources (lead); Supervision (lead); Writing – original draft (supporting); Writing – review & editing (equal).

## Supporting information

Appendix S1

## Data Availability

Data are archived and openly available on DRYAD: https://doi.org/10.5061/dryad.8gtht76r7.
