# Supplementary material for: Trophic niche partitioning between two prey and their incidental predators revealed various threats for an endangered species
Source: Ecol Evol. 2022 Mar 18;12(3):e8742. doi: 10.1002/ece3.8742 (PMC8933322; doi:10.1002/ece3.8742)
Supplement: Supplementary file 1 — Appendix S1 [file ECE3-12-e8742-s001.docx]

**APPENDIX S1**

**Trophic niche partitioning between two prey and their incidental predators revealed various threats for an endangered species**

Ève Rioux, Fanie Pelletier & Martin-Hugues St-Laurent

**Plant sample collection**

We collected a few plant species per plant functional group: aquatic plants (3 *Nuphar variegatum*), deciduous trees (3 *Betula papyrifera*, 9 *Sorbus americana,* 9 *Salix spp.,* 3 *Populus balsamifera*), ericaceous shrubs (6 *Vaccinium spp,* 3 *Kalmia augustifolia,* 3 *Ledum groenlandicum*), evergreen trees (9 *Picea glauca, 9 Abies balsamea*), ferns (3 *Dryopteris spp.,* 6 *Athyrium filix-femina*), forbs (6 *Taraxacum spp.,* 3 *Hieracium pretense,* 3 *Maianthemum canadense,* 6 *Clintonia borealis,* 3 *Juncus spp.,* 9 *Epilobium angustifolium,* 3 *Aralia nudicaulis,* 6 *Cornus canadensis,* 9 *Trientalis borealis,* 3 *Ranunculus septentrionalis,* 6 *Trifolium spp.,* 3 *Fragaria Americana,* 3 *Vicia cracca*), fungi (5 *Agaricaceae* family), graminoids (2 *Carex spp*., 3 *Scirpus spp*., 4 *Agrostis borealis,* 3 *Festuca obtuse,* 6 *Habenaria dilatata*), horsetails (*9* *Equisetum arvense*), and shrubs (9 *Alnus spp.,* 6 *Cornus stolonifera,* 3 *Amelanchier bartramiana,* 9 *Rubus idaeus,* 9 *Ribes spp.,* 6 *Lonicera canadensis,* 3 *Diervilla lonicera,* 6 *Viburnum spp.,* 6 *Acer spicatum*).

**Food sources selection**

The food sources that we included in the Bayesian stable isotope mixing models for the three species studied (moose, bears and coyotes) were chosen based on studies conducted in our study area and literature reviews. Briefly, DNA barcoding analyses of moose scats conducted in the same study area as ours observed a consumption of deciduous trees, evergreen trees, and shrubs in summer and autumn (Christopherson et al., 2019). Consumption of shrub and tree leaves is also reported in other moose populations in Quebec (Drucker et al., 2010; Dussault et al., 2004) while consumption of ferns by moose is reported in Maine (Lautenschlager et al., 1997) and Alaska (Welch et al., 2015). Few studies described the diet of coyotes and black bears in our study area based on scat analyses and also in other adjacent regions. Coyote scat analyses conducted in Gaspésie (Boisjoly et al., 2010), in the adjacent eastern New Brunswick (Dumond et al., 2001) and on the south shore of St-Lawrence River in southeastern Quebec (Tremblay et al., 1998) reported a consumption of deer, moose, hare, fruits, and graminoids by coyotes. Boisjoly et al. (2010) found caribou hair in six coyote faeces on 150 analysed. Black bears are opportunistic omnivores, feeding on invertebrates, plants, vertebrates, and also on anthropogenic resources (Baldwin & Bender, 2009; Boileau, 1993; Boisjoly et al., 2010; Gaudry, 2013; Lesmerises et al., 2015; Mosnier et al., 2008). Diet composition also vary significantly with resource and habitat availability (Brodeur et al., 2008; Latham et al., 2011). In our study area, Boileau (1993) and Mosnier et al. (2008) analysed 265 black bear scats and reported that black bears consumed mainly plants, such as graminoids, dandelions, horsetails, willow, and fruits. They also reported consumption of moose fawn, hare, and beaver (*Castor Canadensis*), but no trace of caribou consumption was found in the 265 faeces analysed. Crête and Desrosiers (1995) reported caribou consumption by coyotes and black bears in Gaspésie.

**Bayesian priors**

We included informative prior distributions on the dietary proportions to increase the robustness of the mixing models (Moore & Semmens, 2008; Stock & Semmens, 2016) based on studies conducted in our study area. For moose, priors of diet composition came from Christopherson et al. (2019) study that analysed 40 fecal pellets using DNA barcoding analyses, while for predators, we used data from scat analyses from 60 black bear faeces and from 115 coyote faeces (Martin-Hugues St-Laurent, *unpublished data*). Specific priors were reported in Table S2.

**REFERENCES**

Baldwin, R. A., & Bender, L. C. (2009). Foods and nutritional components of diets of black bear in Rocky Mountain National Park, Colorado. *Canadian Journal of Zoology*, *87*(11), 1000-1008.

Boileau, F. (1993). Utilisation de l’habitat par l’ours noir (*Ursus americanus*) dans le parc de conservation de la Gaspésie (M.Sc. thesis). Université Laval.

Boisjoly, D., Ouellet, J.-P., & Courtois, R. (2010). Coyote habitat selection and management implications for the Gaspésie caribou. *Journal of Wildlife Management*, *74*(1), 3–11. doi: 10.2193/2008-149

Boudreau, F., 1981. Écologie des étages alpin et subalpin du Mont Jacques-Cartier, parc de la Gaspésie, Québec (M.Sc. thesis). Université Laval, Québec.

Brodeur, V., Ouellet, J. P., Courtois, R., & Fortin, D. (2008). Habitat selection by black bears in an intensively logged boreal forest. *Canadian Journal of Zoology*, *86*(11), 1307–1316. doi: 10.1139/Z08-118

Calenge, C., 2019. Home range estimation in R: The adehabitatHR package. Retrieved from <https://cran.r-project.org/web/packages/adehabitatHR/vignettes/adehabitatHR.pdf>

Crête, M., & Desrosiers, A. (1995). Range expansion of coyotes, *Canis latrans*, threatens a remnant herd of caribou, *Rangifer tarandus*, in Southeastern Québec. *Canadian Field-Naturalist*, *109*, 227–235.

Christopherson, V., Tremblay, J.-P., Gagné, P. N., Bérubé, J., & St-Laurent, M.-H. (2019). Meeting caribou in the alpine: Do moose compete with caribou for food? *Global Ecology and Conservation*, *20*, e00733. doi: 10.1016/j.gecco.2019.e00733

Drucker, D. G., Hobson, K. A., Ouellet, J.-P., &Courtois, R. (2010). Influence of forage preferences and habitat use on ^13^C and ^15^N abundance in wild caribou (*Rangifer tarandus caribou*) and moose (*Alces alces*) from Canada. *Isotopes in Environmental and Health Studies, 46*(1), 107–121.

Dumond, M., Villard, M.-A., & Tremblay, É. (2001). Does coyote diet vary seasonally between a protected and an unprotected forest landscape? *Ecoscience*, *8*(3), 301–310. doi: 10.1080/11956860.2001.11682657

Dussault, C., Courtois, R., Ouellet, J.-P., Huot, J., & Breton, L. (2004). Effet des facteurs limitatifs sur la sélection de l’habitat par l’orignal : une étude de trois ans dans le parc de la Jacques-Cartier. *Le Naturaliste Canadien, 128*(2), 38–45.

Gaudry, W. (2013). *Impact des structures anthropiques linéaires sur la sélection d’habitat du caribou, de l’ours noir et du coyote en Gaspésie* (M.Sc. thesis). Université du Québec à Rimouski.

Latham, A. D. M., Latham, M. C., & Boyce, M. S. (2011). Habitat selection and spatial relationships of black bears (*Ursus americanus*) with woodland caribou (*Rangifer tarandus caribou*) in northeastern Alberta. *Canadian Journal of Zoology*, *89*(4), 267-277.

Lautenschlager, R. A., Crawford, H. S., Stokes, M. R., & Stone, T. L. (1997). Forest disturbance type differentially affects seasonal moose forage. *Alces*, *33*(1974), 49–73.

Lesmerises, R., Rebouillat, L., Dussault, C., & St-Laurent, M.-H. (2015). Linking GPS telemetry surveys and scat analyses helps explain variability in black bear foraging strategies. *PLoS ONE*, *10*(7), 1–14. doi: 10.1371/journal.pone.0129857

Moore, J. W., & Semmens, B. X. (2008). Incorporating uncertainty and prior information into stable isotope mixing models. *Ecology Letters*, *11*(5), 470–480. doi: 10.1111/j.1461-0248.2008.01163.x

Mosnier, A., Ouellet, J.-P., & Courtois, R. (2008). Black bear adaptation to low productivity in the boreal forest. *Ecoscience*, *15*(4), 485–497. doi: 10.2980/15-4-3100

Stock, B. C., & Semmens, B. X. (2016). Unifying error structures in commonly used biotracer mixing models. *Ecology*, *97*(10), 2562–2569.

Tremblay, J.-P., Crête, M., & Huot, J. (1998). Summer foraging behaviour of eastern coyotes in rural versus forest landscape: A possible mechanism of source-sink dynamics. *Ecoscience*, *5*(2), 172–182. doi: 10.1080/11956860.1998.11682456

Welch, J. H., Barboza, P. S., Farley, S. D., & Spalinger, D. E. (2015). Nutritional value of habitat for moose on urban and military lands. *Journal of Fish and Wildlife Management*, *6*(1), 158–175. doi: 10.3996/062014-JFWM-045


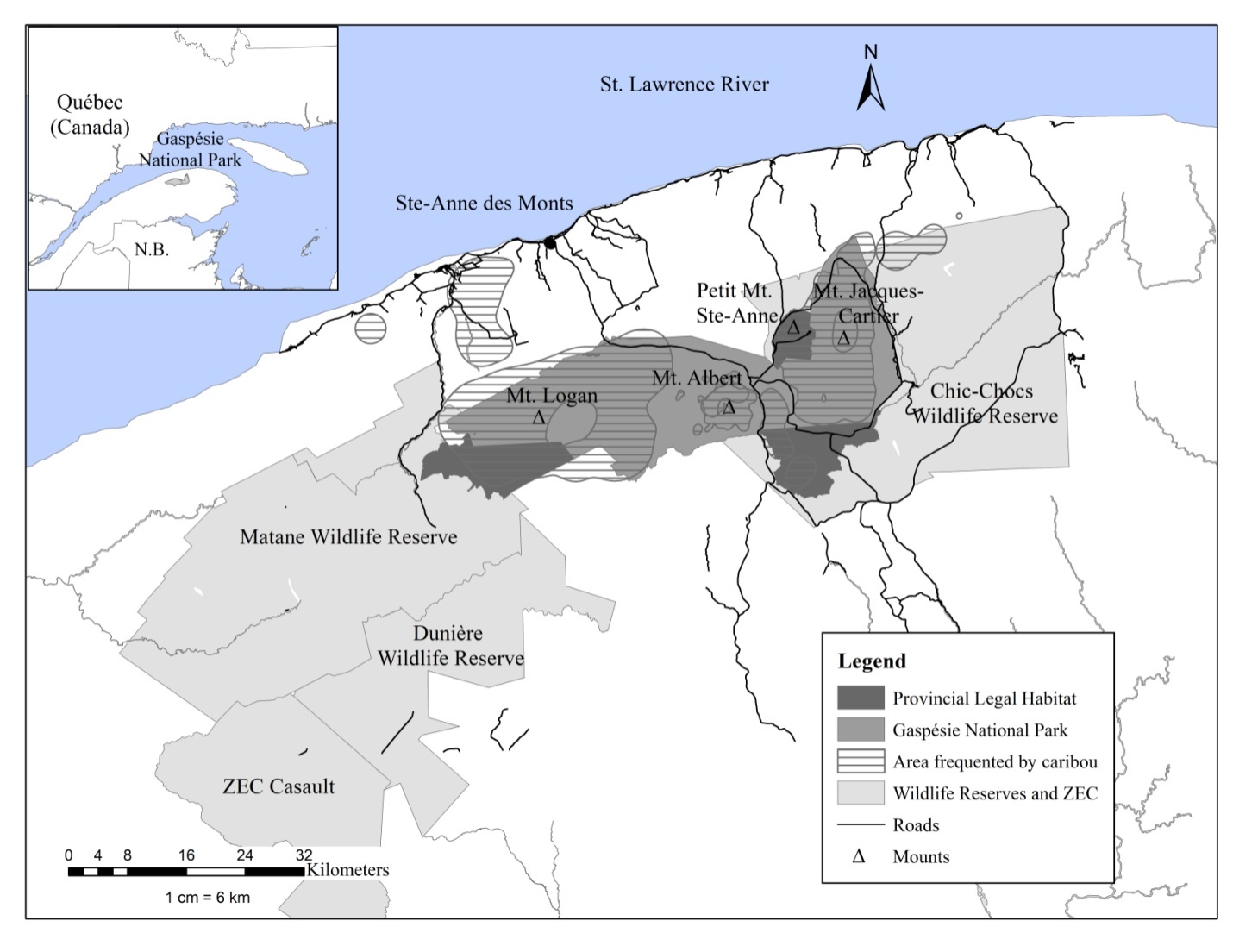


**Figure S1.** Study area of the Atlantic-Gaspésie caribou, moose, coyote and black bear populations in the Gaspésie National Park and the surrounding Wildlife Reserves and controlled harvesting zone (ZEC), Québec, Canada (Mount Logan: 1 128 m, Mount Albert: 1 154 m, Mount Jacques-Cartier: 1 268 m, and Petit Mount Ste-Anne: 1 147 m). The study area is composed of three distinct vegetation zones: the montane boreal forest belt (100-900m) is mainly composed of balsam fir, white spruce (*Picea glauca*), black spruce (*P. mariana*), and white and yellow birch (*Betula alleghaniensis*), the transitional subalpine zone area (900-1050m) is a krummholz belt characterized by stunted trees and shrubs, and the alpine tundra (˃1050m) is mainly composed of lichens, mosses, graminoids and ericaceous shrubs shaped by the exposure to wind (Boudreau, 1981). The area frequented by caribou was estimated using a 95% kernel density estimator (kernelUD, package adehabitatHR, Calenge 2019) using GPS locations of 43 Gaspésie caribou collected from a telemetry monitoring program that took place between 2013 and 2016. Three main summits are used by this caribou population; from west to east, the Logan ridge, the Albert plateau and the McGerrigle area.

**Table S1.** Means and standard deviations of carbon (C) and nitrogen (N) concentrations measured in dietary predator food sources.

| **Food sources** | **C (mg)** | | **N (mg)** | |
| --- | --- | --- | --- | --- |
|  | **mean** | **SD** | **mean** | **SD** |
| Animal |  |  |  |  |
| Caribou | 0.29 | 0.06 | 0.09 | 0.02 |
| Deer | 0.45 | 0.05 | 0.15 | 0.02 |
| Grouse | 0.47 | 0.01 | 0.16 | 0.01 |
| Hare | 0.47 | 0.02 | 0.16 | 0.01 |
| Insects | 0.51 | 0.06 | 0.12 | 0.02 |
| Large rodents | 0.48 | 0.03 | 0.16 | 0.01 |
| Moose | 0.50 | 0.04 | 0.17 | 0.01 |
| Small rodents | 0.48 | 0.03 | 0.16 | 0.01 |
| Plant |  |  |  |  |
| Dandelion | 0.26 | 0.02 | 0.02 | 0.00 |
| Fruits | 0.29 | 0.03 | 0.02 | 0.01 |
| Graminoid | 0.29 | 0.02 | 0.02 | 0.00 |
| Other plants | 0.30 | 0.04 | 0.01 | 0.01 |
| Willow | 0.32 | 0.04 | 0.02 | 0.01 |

**Table S2.** Informative priors on the dietary proportions included in the mixing models for the three species studied.

| **Food sources** | **Priors** | | | |
| --- | --- | --- | --- | --- |
|  | **Moose** | **Coyote** | **Bear** |  |
| Animal |  |  |  |  |
| Caribou |  | 0.03 | 0.01 |  |
| Deer |  | 0.44 | 0.12 |  |
| Grouse |  | 0.006 | 0.02 |  |
| Hare |  | 0.11 | 0.03 |  |
| Insects |  | 0.003 | 0.04 |  |
| Large rodents |  | 0.03 |  |  |
| Moose |  | 0.27 | 0.06 |  |
| Small rodents |  | 0.02 |  |  |
| Plant |  |  |  |  |
| Aquatic plants |  |  |  |  |
| Deciduous trees | 0.65 |  |  |  |
| Ericaceous | 0.03 |  |  |  |
| Evergreen trees | 0.07 |  |  |  |
| Ferns |  |  |  |  |
| Forbs | 0.01 |  |  |  |
| Fungi |  |  |  |  |
| Graminoids | 0.01 |  |  |  |
| Horsetails | 0.02 |  |  |  |
| Shrubs | 0.17 |  |  |  |
| Fruits & Graminoids |  | 0.17 |  |  |
| Fruits & Graminoids & Dandelions |  |  | 0.66 |  |
| Willow |  |  | 0.02 |  |

**Code source example for black bear mixing model**

#######################

# SIMM ANALYSIS

library(simmr)

library(siar)

library(viridis)

# Load your data

# Set the working directory

setwd('C:/Users/Eve/Documents/Doctorat/These/Chapitre 4 - Niche partitioning/Analyses/Simmr/Simmr Pred')

#######################

# HAIR - BEARS

# Read in consumers

consumers = read.table('Ours.txt',header=TRUE)

as.matrix(consumers)

head(consumers)

# Read in sources

sources = read.table('SourcesO2.txt',header=TRUE)

head(sources)

# Read in TEFs

corrections = read.table('TefO2.txt',header=TRUE)

head(corrections)

# Read in Conc Dependance

concdepdemo = read.table('ConcDepOur2.txt',header=TRUE)

head(concdepdemo)

# Load the data

simmr_ours = simmr_load(mixtures = as.matrix(consumers[,c(2,1)]),

source_names = as.character(sources[,1]),

source_means = as.matrix(sources[,c(4,2)]),

source_sds = as.matrix(sources[,c(5,3)]),

correction_means = as.matrix(corrections[,c(4,2)]),

correction_sds = as.matrix(corrections[,c(5,3)]),

concentration_means = as.matrix(concdepdemo[,c(4,2)]))

# Plot the isospace plot

plot(simmr_ours, xlab=expression(paste(delta^13, "C (\u2030)",sep="")),

ylab=expression(paste(delta^15, "N (\u2030)",sep="")),

title='', mix_name='Bears', ggargs = theme(legend.position = 'None', axis.text=element_text(size=23), axis.title=element_text(size=23),panel.grid.major = element_blank(), panel.grid.minor = element_blank(), panel.background = element_blank(), axis.line = element_line(colour = "black"))) + xlim(-32,-17) + ylim(-1, 13)

ggsave("Biplot Ours.jpeg", width = 11, height = 8, dpi = 600)

######### Priors

# Prior information from 60 fecal contents (Martin-Hugues St-Laurent *unpublished data*)

# We can’t put 0

proportion_means = c(0.02,0.12,0.02,0.03,0.04,0.10,0.62,0.02)

# ...and proportion standard deviations:

proportion_sds = c(0.1,0.1,0.1,0.1,0.1,0.1,0.1,0.1)

# We put this into the simmr_elicit function as follows:

prior = simmr_elicit(8, proportion_means,

proportion_sds)

# This may take a few moments to run as the code tries to optimise the parameters of a prior distribution which matches these means and standard deviations, which sometimes may not be exactly possible.

# When finished, the model can be run using these prior distributions:

simmr_out_informative = simmr_mcmc(simmr_ours,

prior_control=list(means=prior$mean,

sd=prior$sd))

# MCMC run - needs slightly longer

simmr_out_informative = simmr_mcmc(simmr_ours,prior_control=list(means=prior$mean,

sd=prior$sd),

mcmc_control=list(iter=100000,burn=10000,thin=100,n.chain=4))

# Look the convergence, it must be close to 1

summary(simmr_out_informative, type = 'diagnostics')

plot(simmr_out_informative, type = 'matrix')

# The new quantiles are:

summary(simmr_out_informative, type = c('statistics', 'quantiles','correlations'))

plot(simmr_out_informative, type = 'boxplot', ggargs = theme(text=element_text(size=23)), title="")

plot(simmr_out_informative, type = 'boxplot', title=' ', ggargs = theme(text=element_text(size=23))) + ylim(0, 1)

ggsave("Boxplot Ours.jpeg", width = 11, height = 8, dpi = 600)

# COMBINE SOURCES

simmr_out_combine = combine_sources(simmr_out_informative2,

to_combine=c('Fruits','Gram','Dandelion'),

new_source_name='FruitsGramDan')

plot(simmr_out_combine$input,xlab=expression(paste(delta^13, "C (\u2030)",sep="")),

ylab=expression(paste(delta^15, "N (\u2030)",sep="")),

title='', mix_name='Bears', ggargs = theme_classic(base_size = 23))

plot(simmr_out_combine,type='boxplot',ggargs = theme(text=element_text(size=23)), title="")

plot(simmr_out_combine,type='matrix',title='simmr output: combined sources')

plot(simmr_out_combine,type='density',title='simmr output: combined sources')

plot(simmr_out_combine,type='histogram',title='simmr output: combined sources')

summary(simmr_out_combine, type = c('statistics', 'quantiles','correlations'))
